# Maternal Th17 Profile after Zika Virus Infection Is Involved in Congenital Zika Syndrome Development in Children

**DOI:** 10.3390/v15061320

**Published:** 2023-06-04

**Authors:** Eder M. S. Fialho, Emanoel M. Veras, Caroline M. de Jesus, Líllian N. Gomes, Ricardo Khouri, Patrícia S. Sousa, Marizélia R. C. Ribeiro, Rosângela F. L. Batista, Luciana C. Costa, Flávia R. F. Nascimento, Antônio A. M. Silva, Paulo V. Soeiro-Pereira

**Affiliations:** 1 Health Sciences Graduate Program, Biological and Health Sciences Center, Federal University of Maranhão, São Luís 65080-805, MA, Brazil; eder.fialho@discente.ufma.br (E.M.S.F.); flavia.nascimento@ufma.br (F.R.F.N.); paulo.soeiro@ufma.br (P.V.S.-P.); 2 Medical School, Federal University of Maranhão, São Luís 65080-805, MA, Brazil; emanoel.veras@discente.ufma.br; 3 Health and Technology Graduate Program, Biological and Health Sciences Center, Federal University of Maranhão, São Luís 65080-805, MA, Brazil; caroline.mj@discente.ufma.br; 4 Department of Immunology, University of São Paulo, São Paulo 05508-000, SP, Brazil; lillian.gomes@usp.br; 5 Gonçalo Moniz Research Institute, FIOCRUZ-Bahia, Salvador 40296-710, BA, Brazil; ricardo.khouri@fiocruz.br; 6 Reference Center on Neurodevelopment, Assistance and Rehabilitation of Children/NINAR—State Department of Health of the State of Maranhão, São Luís 65077-357, MA, Brazil; cdneuropatricia@gmail.com; 7 Department of Medicine III, Federal University of Maranhão, São Luís 65020-240, MA, Brazil; 8 Department of Public Health, Federal University of Maranhão, São Luís 65020-060, MA, Brazil; rosangela.flb@ufma.br (R.F.L.B.); luciana13cavalcante@gmail.com (L.C.C.); silva.antonio@ufma.br (A.A.M.S.); 9 Department of Pathology, Federal University of Maranhão, São Luís 65065-545, MA, Brazil

**Keywords:** vertical transmission, microcephaly, disease infection, interleukin-17

## Abstract

Brazil is one of the countries that experienced an epidemic of microcephaly and other congenital manifestations related to maternal Zika virus infection which can result in Congenital Zika Syndrome (CZS). Since the Zika virus can modulate the immune system, studying mothers’ and children’s immune profiles become essential to better understanding CZS development. Therefore, we investigated the lymphocyte population profile of children who developed CZS and their mothers’ immune response in this study. The study groups were formed from the Plaque Reduction Neutralization Test (PRNT) (CZS+ group) result. To evaluate the lymphocyte population profile, we performed phenotyping of peripheral lymphocytes and quantification of serum cytokine levels. The immunophenotyping and cytokine profile was correlated between CSZ+ children and their mothers. Both groups exhibited increased interleukin-17 levels and a reduction in the subpopulation of CD4+ T lymphocytes. In contrast, the maternal group showed a reduction in the population of B lymphocytes. Thus, the development of CZS is related to the presence of an inflammatory immune profile in children and their mothers characterized by Th17 activation.

## 1. Introduction

Zika is an arbovirus belonging to the family Flaviviridae and transmitted by the mosquito of the genus *Aedes*. Infection with this virus causes the disease known as Zika or Zika Virus Fever. The person with Zika can present with an asymptomatic form or a variety of symptoms such as arthralgia, edema of the extremities, low fever, headache, retroorbital pain, conjunctival hyperemia, and maculopapular rash, usually associated with pruritus [1,2].

At the end of 2015, an epidemic of childbirths presenting congenital malformations in Brazil was associated with maternal infection with the Zika virus (ZIKV), resulting in the identification of Congenital Zika Syndrome (CZS) [3,4]. The clinical symptoms of CZS comprised microcephaly, ventriculomegaly, calcifications, arthrogryposis, and cutis veticis gyrata [5]. The neurotropic nature of the virus caused a more severe range of abnormalities affecting the central nervous system [6,7]. This outbreak raised significant concerns about the transmission and pathogenesis of the Zika virus and highlighted the need for preventive measures and research into CZS [8]. Previous studies showed that ZIKV could cause these symptoms by the ability to replicate successfully in neural cells, as demonstrated in an in vitro model (U87-MG lineage). One of the main consequences of this replication is inflammation and cell death [9].

Unlike other congenital syndromes caused by pathogens of the TORCHS group (Toxoplasmosis, Rubella, Cytomegalovirus, Herpes Simplex, and Syphilis), the ZIKV-induced inflammation is less intense, and the mechanisms responsible for triggering the congenital syndrome are still unknown [10]. Furthermore, the variation in maternal manifestations makes it challenging to determine factors related to the child’s susceptibility to the development of CZS [11]. Another issue is the diagnosis in pregnant women and their infants. Due to the long-lasting presence of antibodies against the ZIKV following infection, serology tests do not differentiate between recent and previous infections. Furthermore, the antibodies for Zika and dengue viruses can cross-react, posing a challenge in accurately determining which virus is responsible for the current illness [3,8]. In addition, the pathophysiological and immunoinflammatory mechanisms involved in the teratogenic effects of ZIKV are still indeterminate [12,13].

The inflammation triggered by the Zika virus infection plays a crucial role in the degeneration of nerve cells [14]. Initially believed to be caused by virus-induced apoptosis, the tissue damage may be associated with the immune response rather than the virus itself [15,16]. Although activated leukocytes play a crucial role in eliminating pathogens during viral infections, multiple studies have demonstrated that an uncontrolled response can lead to damage caused by the production of mediators such as tumor necrosis factor-alpha and interleukins-1, 6, and 17 [17,18]. Therefore, regulating the immune response is crucial to prevent excessive tissue damage in response to ZIKV infections. Within this context, microglia infected with ZIKV produced high titers of inflammatory cytokines, including IL-6, MCP-1, TNF-α, IL-1, and IL-8 [19]. Furthermore, the results of in vitro infection of monocytes with different strains of the ZIKV show the virus’s ability to direct innate immune response to a pro-inflammatory profile [20].

Limited studies have examined the link between the immune response of mothers and the development of Congenital Zika Syndrome [21,22]. However, the maternal immune response is closely tied to the outcome of the fetus’s intrauterine exposure to the Zika virus. Therefore, our study aimed to investigate the relationship between the immunological profile of mothers and the development of CZS in their children. By understanding these pathways, we can identify markers that predict the susceptibility and degree of sequelae caused by the syndrome. Such knowledge could be crucial in developing targeted interventions to mitigate the impact of CZS on affected families.

## 2. Materials and Methods

### 2.1. Study Design

The study was conducted in collaboration with the Reference Center for Neurodevelopment, Assistance, and Rehabilitation of Children (NINAR) and comprised a prospective cohort of children and their mothers living in Maranhão, northeastern Brazil.

The study enrolled a prospective cohort of children aged 20 months who had undergone cranial imaging examination showing at least one of the following abnormalities: agenesis/dysgenesis of the corpus callosum, trunk malformation/hypoplasia, reduced cerebral parenchyma volume, ventriculomegaly, calcifications, cortical developmental malformation, and cerebellar malformation/hypoplasia [23]. The cohort excluded children with other congenital syndromes, such as positive tests for TORCHS or neural tube defects.

Out of the 166 children who were referred to the Reference Center for Neurodevelopment, Assistance, and Rehabilitation of Children (NINAR) for investigation of Congenital Zika Syndrome (CZS), 103 underwent PRNT50 testing from July 2017 to July 2018. Of the 63 children who tested negative for PRNT, 30 were included in the control group (CZS−) and showed no signs or symptoms of CZS. Among the 40 children confirmed to have CZS through PRNT testing (CZS+), we collected samples from 27 children to conduct the experiments in this work.

Together with a positive PRNT (at 20 months of age), the presence of alterations in the cranial imaging exam (calcifications, reduction in the volume of the cerebral parenchyma, ventriculomegaly, malformation of the cortical development, malformation/hypoplasia of the cerebellum, malformation/hypoplasia of the brainstem and agenesis/dysgenesis of the corpus callosum) constituted the inclusion criteria in the CZS+ group [23]. Children with alternative causes of congenital syndrome (e.g., positive TORCHS) were excluded, and Children with negative PRNT and cranial alterations were not considered for the control group. Children without suggestive clinical features and those that were PRNT negative formed the CZS− group.

The mothers’ clinical data were collected through a structured questionnaire demonstrating the main clinical manifestations during the infection: skin rash (65%), headache (54%), fever (53%), and pruritus (50%). In addition to clinical data, maternal blood was collected simultaneously with the sample from children.

### 2.2. PRNT (Plaque Reduction Neutralization Test)

PRNT testing played a crucial role in characterizing the studied populations concerning their exposure to the virus and the resulting immune/inflammatory response maintenance. The neutralizing capacity of serum samples against the Zika virus was determined using the plaque reduction neutralization test (PRNT), as previously described [24], with some modifications to the established protocol [25]. The PRNT50 was set at a cut-off value of 50%, indicating the highest serum dilution (ranging from 1:8 to 1:4096) required to inhibit 50% of the virus-induced plaque formation. Vero cells (2 × 105 cell/well) and the Brazilian ZIKV PE/243 strain (100 PFU/well) were used. Diluted serum samples (1:1) in Dulbecco’s Modified Eagle’s medium (DMEM, Gibco, New York, NY, USA) with a 2% fetal bovine serum (FBS, Gibco, New York, NY, USA) and a 1% penicillin/streptomycin were incubated with virus suspension for 60 min at 37 °C. This serum–virus mix was added to the Vero cells and set for another 60 min. Afterward, 300 µL of a 0.6% agarose solution was added, and the plates were re-incubated under identical culture conditions for five days. The reactions were finally revealed using a 2% Naphthol Blue Black solution (Sigma-Aldrich, St. Louis, MO, USA). A PRNT50 value of ≥10 was considered positive.

### 2.3. Immunophenotyping Analysis of Lymphocyte Populations

To characterize the children (at 20 months of age) and their mothers in regard to the profile of T (helper and cytotoxic) and B lymphocytes, we used total leukocytes obtained from 100 µL of hemolyzed peripheral blood with an RBC Lysis solution (QIAGEN, Hilden, Germany). After this step, the cells were incubated with specific antibodies conjugated to fluorochromes at 4 °C for 30 min. After labeling, the samples were washed with a phosphate-buffered solution and taken for analysis by flow cytometry.

T and B lymphocyte populations were characterized by CD3/CD4/CD8 and CD3/CD19 (Becton, Dickinson, and Company, Franklin Lakes, NJ, USA) panels of specific antibodies, respectively. Data acquisition was performed using a BD FACSCalibur flow cytometer (BD Biosciences, San Diego, CA, USA), and data analysis was performed using FlowJo software (Becton, Dickinson and Company, Franklin Lakes, NJ, USA). The percentage of cells expressing the markers and their expression levels, indirectly determined by the mean fluorescence intensity (MFI), represent the main results used to analyze ZIKV infection repercussions in T and B cell populations.

### 2.4. Cytokine Evaluation by Cytometric Bead Array (CBA)

Detection and quantification of cytokines in plasma were performed using the Cytometric Bead Array (CBA) method described by the manufacturer. The biomarkers evaluated were IL-17, IL-10, IL-6, IL-4, IL-2, TNF-α, and IFN-γ, using a specific laboratory kit (Human CBA Kit Th1 Th2 Th17, Becton, Dickinson, and Company, Franklin Lakes, NJ, USA). Each cytokine’s pg/mL quantification was based on a standard curve with known concentrations. The samples were acquired in a FACS Calibur (BD Biosciences, San Diego, CA, USA).

### 2.5. Data Analysis

The data were tested for normality using the Shapiro–Wilk test and analyzed using Student’s *t*-test and the Mann–Whitney test. Results were reported as mean ± standard deviation, and significance was set at *p* < 0.05. Pearson’s correlation coefficient was used to determine the association between continuous variables. Prism v.8 (GraphPad, Boston, MA, USA) was used to conduct these analyses.

### 2.6. Ethical Aspects

The study adhered to the principles outlined in the Declaration of Helsinki and was subjected to the approval of the Research Ethics Committee of the Federal University of Maranhão Hospital (CAAE: 65897317.1.0000.5086). All participants provided written informed consent before undergoing any study-related procedures. The mothers provided informed consent for participation of themselves and their children. The research team strictly followed the ethical guidelines throughout the study to ensure the safety and well-being of the participants.

## 3. Results

### 3.1. CZS+ Children and Their Mothers Present Similar T and B Lymphocyte Profile

**Figure 1:**
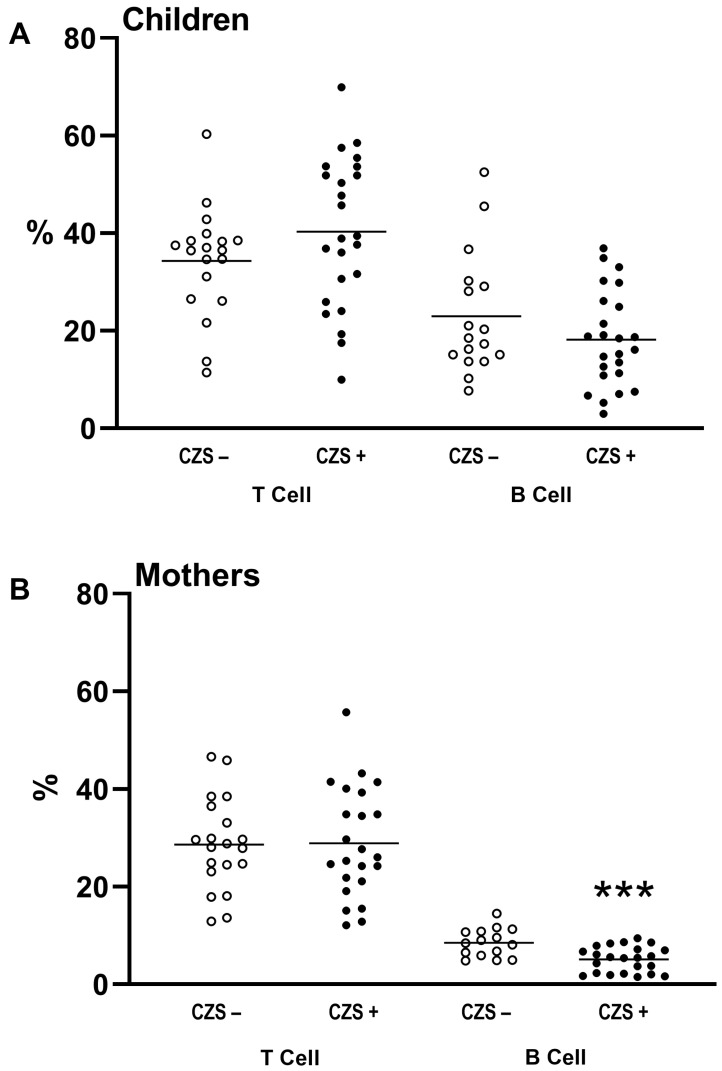
Lower CD3−CD19+ lymphocytes in mothers of CZS+ children compared to those with CZS− negative children. Blood samples were obtained from both mothers and children to determine the percentage of T lymphocytes (CD3+) and B lymphocytes (CD3−/CD19+). The CZS+ group (represented by filled circles, N = 27) and CZS− group (represented by hollow circles, N = 30) were compared both for children (**A**) and mothers (**B**), with each point representing an individual and the bar representing the median of the group. Student’s *t*-test for statistical analysis, with significance indicated by (***) *p* < 0.0001.

**Figure 2:**
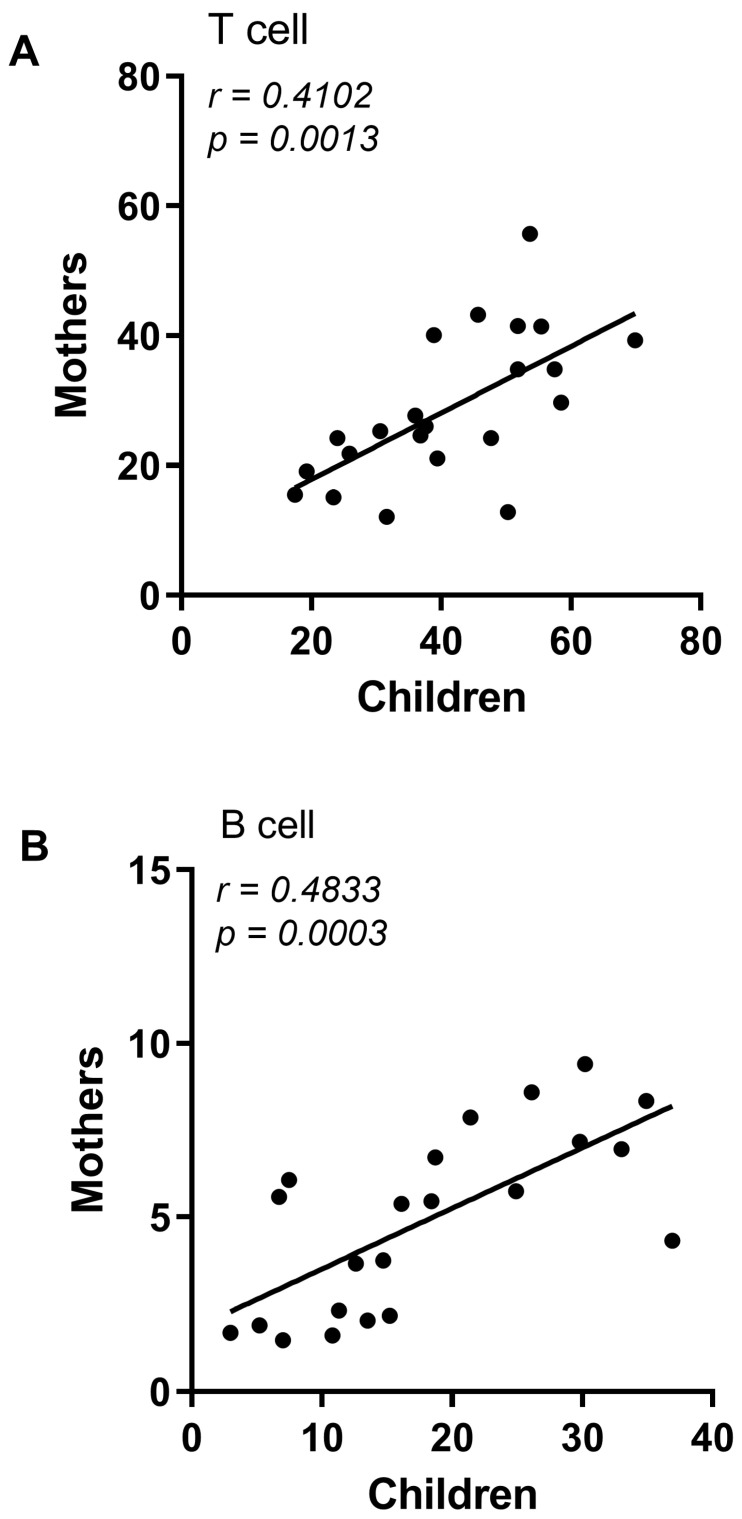
Correlation between Congenital Zika Syndrome (CZS) children’s cell populations and their mothers. (**A**) Correlation between T cells of children and their mothers of the CZS+ group (N = 27). (**B**) Correlation between B cells of children and their mothers of the CZS+ group (N = 27). Each point represents a pair of mother and child, and the line represents the direction of the correlation. Pearson’s two-tailed test was used to build the correlations, *p* < 0.05.

The CZS+ children did not show differences in the percentage of T or B lymphocytes compared to CZS− children (Figure 1A; *p* = 0.1599 and *p* = 0.1717). Regarding mothers, there were no changes in the percentage of T lymphocytes (Figure 1B; *p* = 0.1086). However, there was a decrease in the percentage of B lymphocytes (Figure 1B; *p* = 0.0005) of mothers of children with CZS compared to those without CZS. Furthermore, we found a correlation between the leukocyte profile of mothers with children CZS+ (Figure 2A; *p* = 0.0013; Figure 2B; *p* = 0.0003), which was not found between children CZS− and their mothers (Supplementary Material).

**Figure 3:**
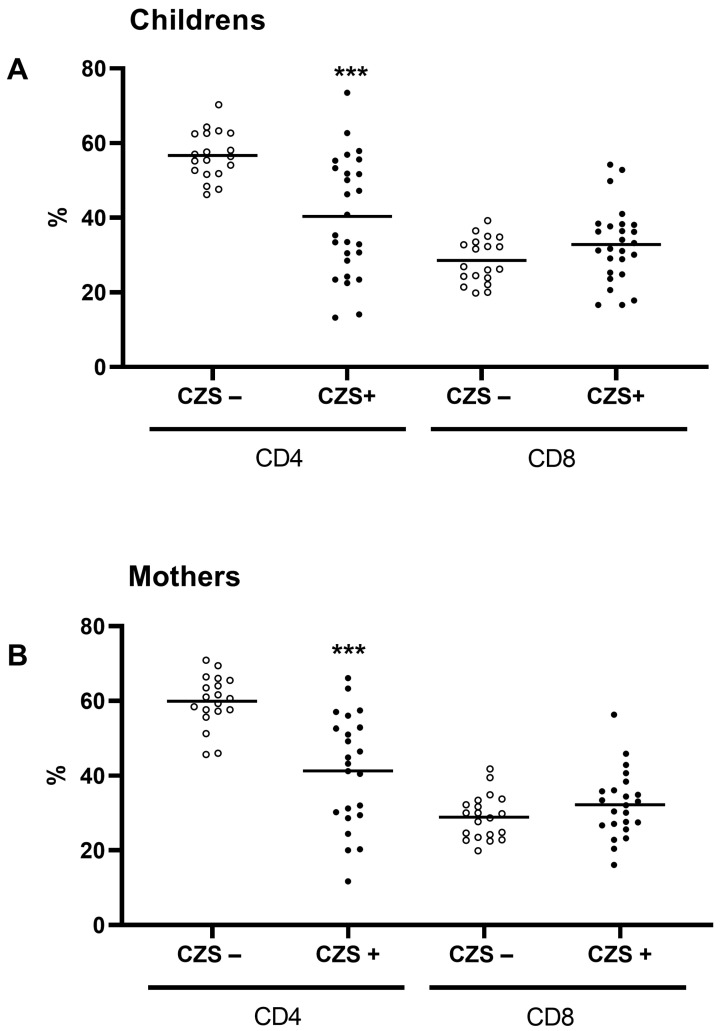
Decrease in the CD3+CD4+ helper T lymphocyte population in children with Congenital Zika Syndrome and in their mothers. Mothers and children had their peripheral blood collected and used to quantify the percentage of helper (CD3+/CD4+) and cytotoxic (CD3+/CD8+) T lymphocytes. The CZS− group (represented by hollow circles) and CZS+ group (represented by filled circles) with 30 and 27 participants, respectively, were compared among children (**A**) and mothers (**B**). Each point represents an individual, and the bar represents the group’s median. Student’s *t*-test was conducted for group comparison, with(***) *p* < 0.0001 as the significant values.

**Figure 4:**
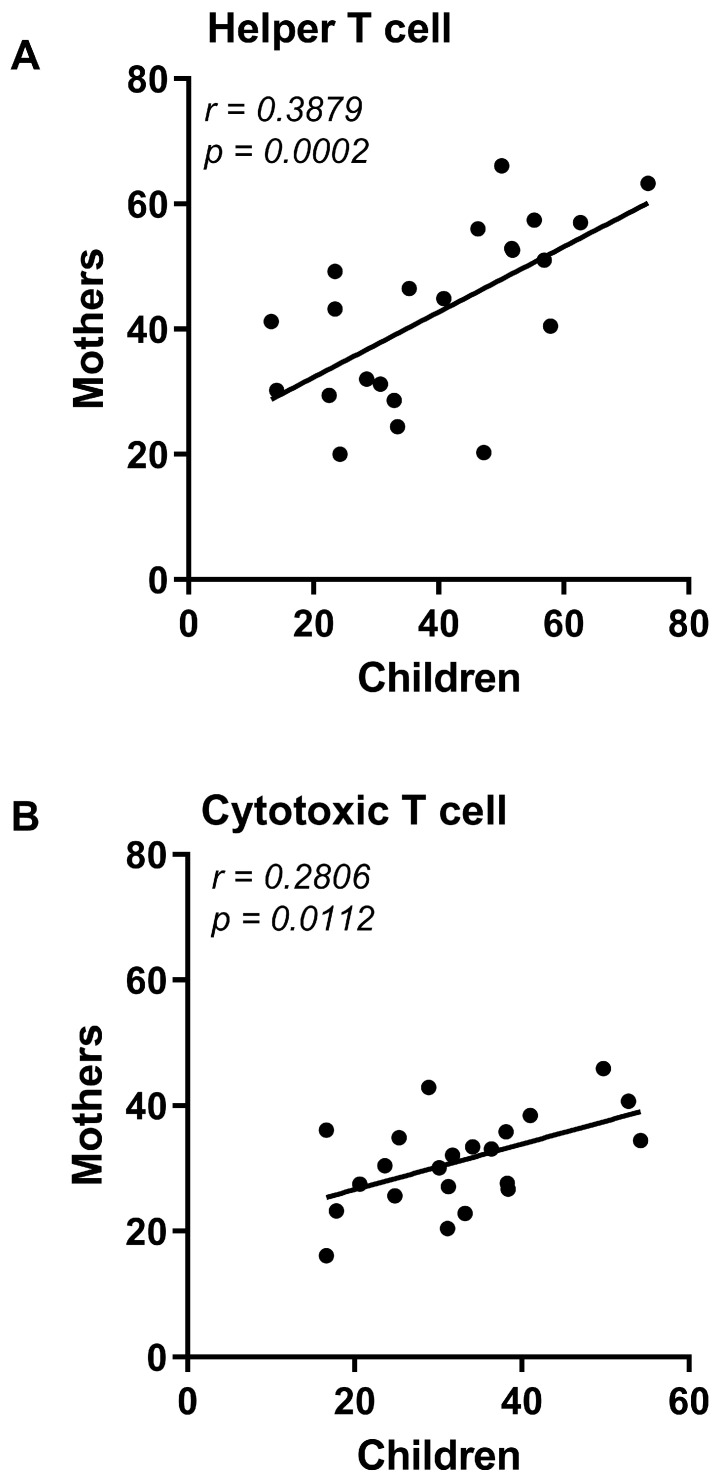
Correlation between Congenital Zika Syndrome (CZS) children’s T lymphocyte subpopulations and their mothers. (**A**) Correlation between CD3+/CD4+ T cells of children and their mothers of the CZS+ group (N = 27). (**B**) Correlation between CD3+/CD8+ T cells of children and their mothers of the CZS+ group (N = 27). Each point represents a pair of mother and child, and the line represents the direction of the correlation. Pearson’s two-tailed test was used to build the correlations, *p* < 0.05.

We further investigated the T lymphocyte subtypes, CD3+/CD4+ and CD3+/CD8+. CZS+ children (Figure 3A) and their mothers (Figure 3B) showed a decrease in the percentage of helper T lymphocytes compared to their respective control groups. However, there was no difference in the rate of cytotoxic T lymphocytes in children (Figure 3A) or their mothers (Figure 3B). Importantly, there was a correlation between the CD4+ and CD8+ values of the CZS+ children and their mothers’ values (Figure 4A,B).

### 3.2. CZS+ Children Present Augmented IL-17 Production

**Figure 5:**
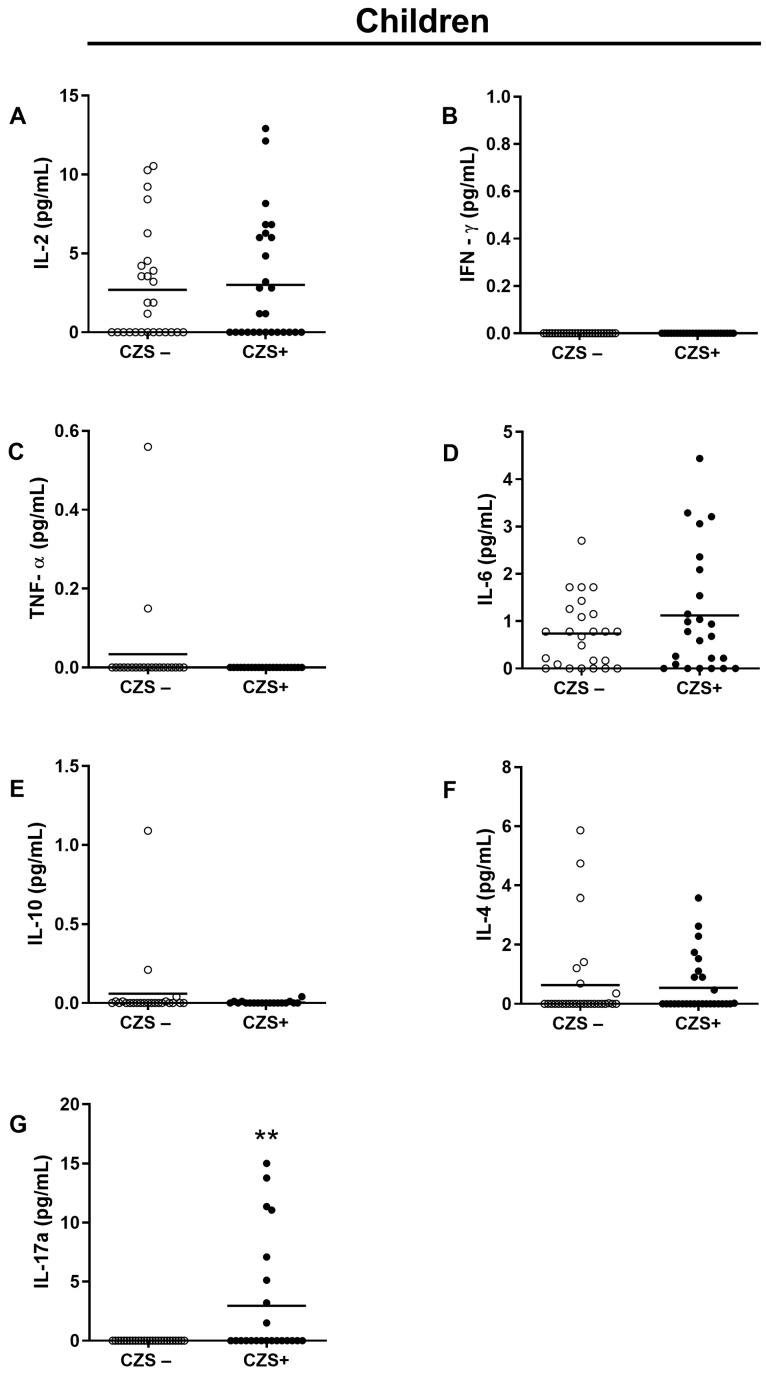
CZS+ children present increased IL-17 in circulation. Peripheral blood plasma was collected from CZS− (N = 30) and CZS+ (N = 27) children. Cytokines IL-2 (**A**), IFN-γ (**B**), TNF-α (**C**), IL-6 (**D**), IL-10 (**E**), IL-4 (**F**) and IL-17 (**G**) were evaluated using plasma samples through the bead capture method (CBA) by flow cytometry analysis. The groups were compared using a Student’s *t*-test, where statistical significance was set at *p* < 0.0001 (**). Each data point on the graph represents an individual, while the bar depicts the median value for the group.

**Figure 6:**
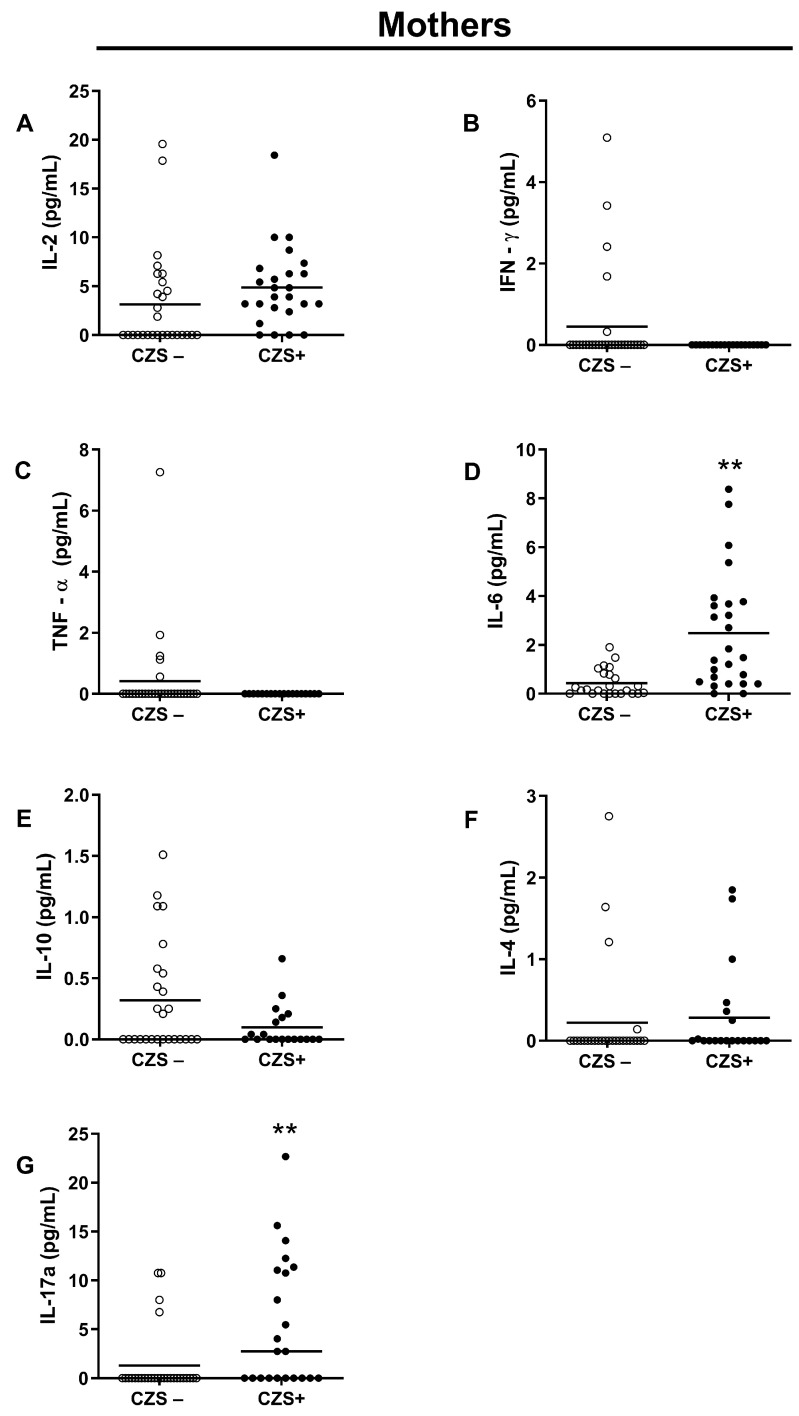
Increased IL-6 and IL-17 in plasma from CZS+ mothers. Peripheral blood plasma was collected from CZS− (N = 30) and CZS+ (N = 27) mothers. Cytokines IL-2 (**A**), IFN-γ (**B**), TNF-α (**C**), IL-6 (**D**), IL-10 (**E**), IL-4 (**F**) and IL-17 (**G**) were evaluated using plasma samples through the bead capture method (CBA) by flow cytometry analysis. The groups were compared using a Student’s *t*-test, where statistical significance was set at *p* < 0.0001 (**). Each data point on the graph represents an individual, while the bar depicts the median value for the group.

There was no difference between the two groups (CZS− and CZS+) concerning plasma IL-2 (Figure 5A; *p* = 0.9021; Figure 6A; *p* = 0.1859), IFN-γ (Figure 5B; *p* = 0.9999; Figure 6B; *p* = 0.1287), TNF-α (Figure 5C; *p* = 0.4878; Figure 6C, *p* = 0.1409), IL-10 (Figure 5E, *p* = 0.5758; Figure 6E; *p* = 0.2526) and IL-4 (Figure 5F; *p* = 0.6313, Figure 6F; *p* = 0.1696). However, the group of CZS+ children showed an increase in the concentration of IL-17 (Figure 5G; *p* = 0.0011). CZS+ mothers presented an increase in the plasma concentration of IL-6 and IL -17 (Figure 6D; *p* = 0.0004; Figure 6G; *p* = 0.0003).

## 4. Discussion

The clinical features of CZS have been extensively studied in children who showed abnormalities at birth, particularly in relation to microcephaly [26,27,28]. However, research has also shown that some children who appear normal at birth may later exhibit changes in anthropometric indices and progress to severe microcephaly, which can result in delayed neurodevelopment [29,30]. These findings suggest that CZS may have a broader range of clinical manifestations than previously thought and underscore the need for continued research to better understand the disease and its long-term effects. Early identification and intervention may be key in mitigating the impact of CZS on affected individuals and their families.

The clinical evaluation of the children investigated in this study has been previously reported in studies [31,32]. During the cohort follow-up, we collected data on all the children in the CZS+ group and observed that they developed severe microcephaly by the age of 24 months, even those who were born with moderate microcephaly or without microcephaly. This indicates that there may have been an ongoing inflammatory response during this period [33,34]. Our study provides evidence of the potential inflammatory mechanisms that could explain the processes leading to this severe clinical condition later on. These findings contribute to a better understanding of CZS and may aid in the development of more effective treatment strategies.

The experimental design of our study was geared towards evaluating the components of the adaptive immune response, considering the time between Zika virus infection and the development of CZS characteristics. As far as we know, this is the first study to demonstrate the association between the adaptive immune response of mothers infected with Zika during pregnancy and the outcome of the Zika virus infection in their children. Our findings shed light on the immune system’s response to the Zika virus infection and its potential impact on fetal development.

Our finding that demonstrates the reduction in the population of helper T lymphocytes and B lymphocytes from mothers suggests a failure to assemble the effector immune response, which can comprise both the cellular immune and the humoral response, which is essential for the neutralization of viral particles [35]. In a similar scenario, Vyas et al. [36] followed a group of mothers who tested positive for the Hepatitis B virus antigen. They observed that mothers in whom the vertical transmission of the virus occurred exhibited a lower frequency of B lymphocytes in peripheral blood, which was associated with low levels of IL-21. Thus, the authors demonstrated that maternal immune factors are associated with the outcome of congenital infection.

Despite investigating aspects related to the outcome of the viral infection, we did not find differences between the population of cytotoxic lymphocytes. Studies have not yet elucidated the role of CD8+ T lymphocytes in the pathogenesis of CZS, indicating antagonistic roles during infection, both in animal models and in patients. In research using resistant mice of the C57/BL6 strain, it was observed that the infected animals developed a robust CD8 response, generating potential memory cells [37]. On the other hand, the CD8 cell infiltrates in the central nervous system are related to neurodegeneration [14]. This phenomenon is less evident in mice lacking IFN, indicating the participation of these components in the nerve injury progression [38]. 

The role of CD8 cells is controversial not only in animal models but also in patients. In brain tissue biopsies from fatal cases of children born with microcephaly, there was an intense inflammatory infiltrate with an increased expression of CD4 T and CD8 T lymphocyte markers [16]. Thus, even though the role of CD8 T lymphocytes in viral infections is well understood, the chronic stimulus can trigger the deregulation of the cytotoxic response and lead to a more severe disease. 

The fact that we found a reduction In the helper T lymphocyte population in mothers and syndromic children was intriguing. These cells play a significant role in directing and regulating the immune response, and its decrease may be related to some deficit in resistance or susceptibility to diseases. However, when we evaluated the functional lymphocyte response, we found a predominance of cytokines of the Th17 pattern, which gives us space to suggest that, despite the reduction in the number of cells, there is a maintenance of an ongoing inflammatory response. 

Viral infections can modulate the immune response during pregnancy by inducing increased levels of inflammatory cytokines. Ornelas et al. [17] showed a miscellany of inflammatory cytokines in the amniotic fluid of nine pregnant women who had Zika and children with CZS. Activating the immune system in the uterine environment may be one of the first factors that can interfere with the fetus’ development [39]. In general, cytokines such as TNF-a, IFN-g, and IL-1b can be associated with teratogenic effects. TNF-a presents the potential to cause neural tube defects, IFN-g is related to impaired placental development following Zika virus infection, and IL-1b can induce preterm labor [40].

The Zika virus Is related to an increase in cytokine IL-17 expression only in pregnant women [28,31]. In the study of infection in non-pregnant women, the virus increased certain cytokine levels, such as IL-6, IL-2, and IFN-g. However, when the infection occurs in pregnant women, there is an increase in the production of IL-10, IL-6, IL-17, TNF-a, IFN-a, and IFN-g [31]. Even during the acute Zika fever phase in adult patients, IL-17 was not found [5]. However, the amniotic fluid of pregnant women infected with the Zika virus presented IL-17 at a concentration 19 times higher than that of the control group. In this scenario of increased IL-17, the fetuses developed microcephaly [28]. Thus, our study shows that the persistence of the inflammatory profile in mothers, probably induced by the virus during pregnancy, is correlated with the children’s inflammatory profile. This finding suggests that maternal immune components can be transferred to the fetus and cause or influence the development of CZS.

Our prior study [25] investigated the oxidative profile of innate cells in the same cohort of mothers and children. Our results showed that mothers had a higher population of neutrophils with an increased ability to generate free radicals than the control group. These findings support those of our current study, as an increase in neutrophil numbers marks a Th17-mediated inflammation response. Consequently, our results suggest that the inflammatory profile of mothers could be related to the onset of Congenital Zika Syndrome. These findings provide insights into the potential mechanisms underlying the pathogenesis of this syndrome, which could help develop effective therapeutic interventions.

Experimental models of maternal immune activation with mice show that viral infections during pregnancy lead to a pathological immune activation dependent on the production of maternal IL-17 by Th17 cells of the decidua and placenta. The IL-17 produced was transferred to the fetus, which showed atypical cortical development. That, in turn, was associated with increased expression of IL-17 receptors in brain tissue induced by double-stranded RNA injection [41]. A chronic inflammatory response sustained by high levels of IL-17 may cause clinical worsening during CZS. In addition to high levels of IL-17, mothers of syndromic children also had high levels of IL-6, a cytokine that acts as a cofactor in the differentiation of Th17 lymphocytes, as well as causing changes in brain development [42,43].

Beyond the effects of cytokines during pregnancy, infants exposed to maternal immune activation are more sensitive to an inflammatory stimulus. The study followed the offspring of rhesus macaques that received [poly(I:C)] intraperitoneally, mimicking a viral infection, for four years. In the first year, the offspring showed high production of inflammatory cytokines, such as IL-1b, IL-6, and IL-12p40. At the age of 4, the offspring continued with a high production of IL-1b but also showed production of Th2 cytokines, in this case, IL-4 and IL-13 [44]. In another experimental mouse study, the authors observed that after maternal immune activation by injection of [poly(I:C)], a second postnatal injection in the offspring induced high levels of IL-6, IL-17a, and IFN-g, in addition to causing necrosis and failure of the unfolded protein response [45]. These studies emphasize that contact with the inflammatory stimulus in the prenatal period can generate an exacerbated pattern of inflammatory response in the offspring, causing damage that manifests late in development. 

Hence, the development of CZS involves the neurotropic effect of the virus, causing apoptosis and a decrease in the proliferative capacity of nerve cells, as already demonstrated by other authors [6,37]. Beyond that, the infection can also be the origin of cytokines with teratogenic effects produced by pathological immune activation in mothers. Due to this maternal immune activation, there is a chronification of the inflammatory response, which in our study was perceived by the presence of IL-17 in children and mothers, even after 20 months. A possible hypothesis that can explain the high levels of this cytokine is the permanence of Th17 memory resident cells in the tissue (Trm17), which is characterized by the production of cytokines with high deleterious capacity, as already characterized in other contexts [46,47,48].

The first association between maternal immune activation and the emergence of neurological disorders came from the observation that children with autism or schizophrenia had a history of exposure to infection during pregnancy, such as the influenza virus and rubella [49]. Regarding the Zika virus, it is possible to observe behavioral changes in offspring with intrauterine exposure to the virus in murine models at the age stages equivalent to human adolescence [50]. Thus, it is reasonable to suggest that CZS may be related to IL-17-dependent maternal immune activation processes.

Our findings suggest a potential link between maternal factors and the likelihood of developing congenital syndromes. Furthermore, our research suggests a strong correlation between the cellular composition of children with syndromes and that of their mothers. This observation supports the theory that maternal immune components are passed on to the fetus, resulting in a similar immune response profile. These findings highlight the importance of considering maternal factors when assessing a child’s risk for congenital syndromes and underscore the complex interplay between maternal and fetal health. Further investigation into the mechanisms behind this association may lead to improved preventative and therapeutic measures for affected families.

## 5. Conclusions

Our findings suggest that the maternal pro-inflammatory immune profile likely initiates and drives the development and progression of Congenital Zika Syndrome. In addition to presenting evidence that maternal factors may be associated with susceptibility to the development of congenital syndromes, we also found that the cellular profile of syndromic children is correlated with the profile of their mothers, which reinforces the transfer of maternal immune components to the fetus to establish this similarity of the immune profile. An appropriate prognostic model for CZS requires integrating immunological analysis and clinical evaluation of patients. Therefore, it is imperative to combine both aspects to ensure accurate identification of the cases and adequate treatment.

## Data Availability

The information presented in this article is currently undergoing bioinformatics analyses and correlation tests as part of a larger project. As a result, the data are not available to the public. Nevertheless, we are pleased to answer any questions about accessing the results of this paper.

## Supplementary Materials

The following supporting information can be downloaded at: https://www.mdpi.com/article/10.3390/v15061320/s1, Figure S1: Correlation between control group children’s (CZS−) cell populations and their mothers. (A) Correlation between T cells of children and their mothers of the CZS− group. (B) Correlation between B cells of children and their mothers of the CZS− group. Each point represents a pair of mother and child, and the line represents the direction of the correlation. Pearson’s two-tailed test was used to build the correlations, *p* < 0.05, n = 30; Figure S2: Correlation between control group (CZS−) children’s T lymphocyte subpopulations and their mothers. (A) Correlation between CD3+/CD4+ T cells of children and their mothers of the CZS− group. (B) Correlation between CD3+/CD8+ T cells of children and their mothers of the CZS− group. Each point represents a pair of mother and child, and the line represents the direction of the correlation. Pearson’s two-tailed test was used to build the correlations, *p* < 0.05, n = 30.
