# Supplementary material for: Maternal Th17 Profile after Zika Virus Infection Is Involved in Congenital Zika Syndrome Development in Children
Source: Viruses. 2023 Jun 4;15(6):1320. doi: 10.3390/v15061320 (PMC10301288; doi:10.3390/v15061320)
Supplement: Supplementary file 1 [file viruses-15-01320-s001.zip › viruses-2348519-supplementary.pdf]

Supplementary Material

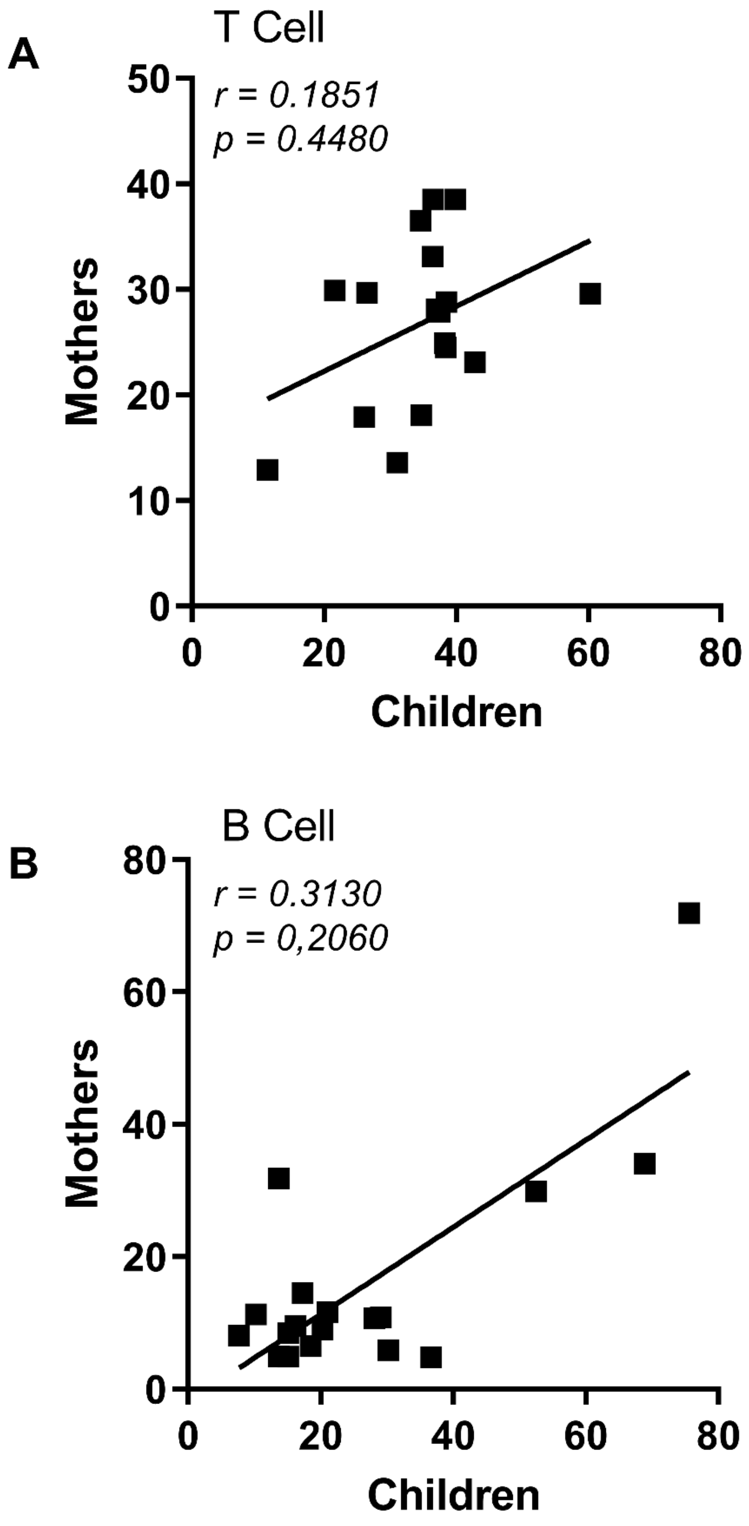

Supplementary figure S1. Correlation between control group children's (CZS–) cell populations and their mothers. (A) Correlation between T cells of children and their mothers of the CZS– group. (B) Correlation between B cells of children and their mothers of the CZS– group. Each point represents a pair of mother and child, and the line represents the direction of the correlation. Pearson's two-tailed test was used to build the correlations, $p < 0.05$, $n = 30$.

A

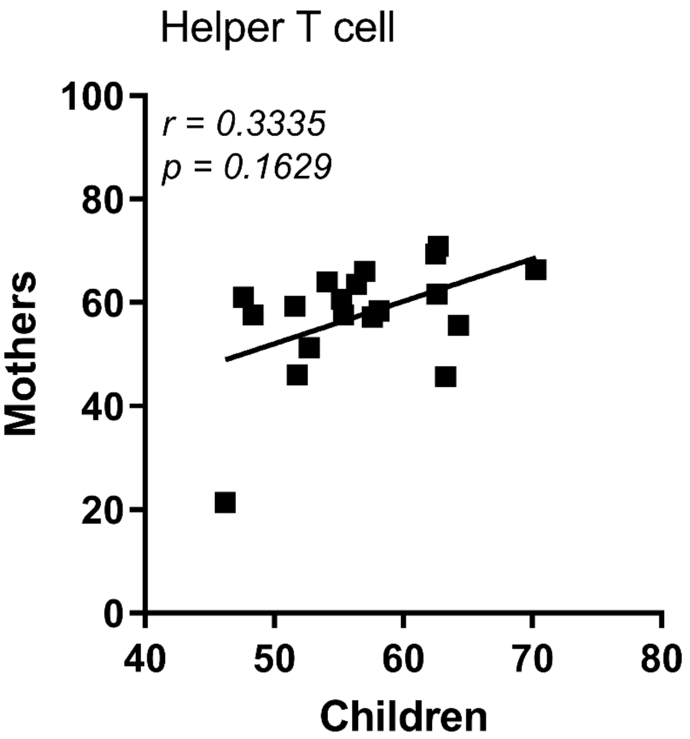

B

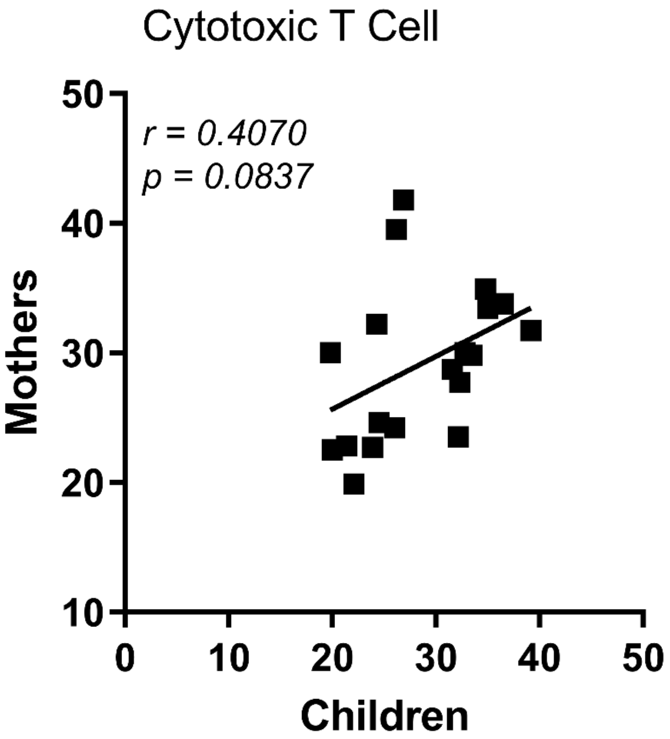

Supplementary figure S2. Correlation between control group (CZS–) children's T lymphocyte subpopulations and their mothers. (A) Correlation between CD3+/CD4+ T cells of children and their mothers of the CZS– group. (B) Correlation between CD3+/CD8+ T cells of children and their mothers of the CZS– group. Each point represents a pair of mother and child, and the line represents the direction of the correlation. Pearson's two-tailed test was used to build the correlations, p <0.05, N = 30.
